# Monitoring infestation levels using an AI-based *Chironomid* quantification tool (CQT) in a laboratory *Xenopus laevis* facility

**DOI:** 10.1186/s13104-026-07919-y

**Published:** 2026-06-19

**Authors:** Maximilian Schwarzbach, Bastian Popper

**Affiliations:** Core Facility Animal Models, Biomedical Center, Medical Faculty, Ludwig-Maximilians-University Munich, Munich, Germany

**Keywords:** Clawed frogs, *Bacillus thuringiensis israelensis*, Diptera, *Chironomidae*

## Abstract

**Objective:**

Insect infestations in laboratory animal facilities represent a potential risk to animal welfare, staff hygiene, and experimental integrity. In this study, we developed and applied a custom AI-assisted image analysis workflow, the *Chironomid* Quantification Tool (CQT-AI), to detect and quantify infestations of non-biting midges (*Chironomidae*) in an aquatic housing system for *Xenopus laevis*. To evaluate the applicability of CQT-AI for monitoring infestation dynamics and treatment effects, different control strategies, including increased water exchange and biological control using *Bacillus thuringiensis israelensis* (Bti), were tested in parallel.

**Results:**

CQT-AI proved to be a reliable tool for detecting and quantifying infestation dynamics of adult *Chironomidae*, enabling objective assessment of treatment effects within 1 min. Increased water exchange resulted in measurable changes in water chemistry but only moderately reduced infestation levels. In contrast, treatment with Bti led to rapid and near-complete eradication of adult midges within six weeks without detectable adverse effects on animal health. Overall, the study demonstrates that CQT-AI provides an efficient and objective approach for monitoring infestation dynamics and evaluating control strategies in aquatic laboratory systems.

## Introduction

Non-biting midges of the family Chironomidae are abundant in freshwater habitats and play key roles in aquatic food webs [1]. They are taxonomically diverse and occupy distinct microhabitats, including running and cold, well-oxygenated waters [2]. Their life cycle comprises egg, larva, and pupa stages in water, followed by free-flying adults [3]. Under controlled laboratory conditions, development can be shortened to 10–21 days due to stable temperatures, nutrient availability, and absence of predators [3–6]. Chironomid proliferation has been reported in wastewater and drinking water systems, where larvae and adults can cause nuisance issues requiring monitoring and control [7, 8]. They may also act as reservoirs for bacteria such as *Aeromonas* and *Vibrio species* [9, 10]. Laboratory animal facilities maintain strict hygiene to prevent microbial introduction and ensure animal health [8, 11]. According to FELASA recommendations, environmental monitoring should include detection of invertebrates as potential biosecurity risks [12]. Clawed frogs are particularly susceptible to waterborne and environmental pathogens [13–15]. Control strategies include mechanical removal, environmental management, and chemical or biological larvicides. Chemical agents such as Methoprene are often unsuitable in laboratory settings due to toxicity and interference with experiments [16–18]. *Bacillus thuringiensis israelensis* (Bti) is a widely used biological control targeting dipteran larvae via Cry and Cyt toxins that disrupt the larval midgut [19–23]. Owing to its low vertebrate toxicity and limited persistence, Bti is considered compatible with aquatic husbandry systems, including those containing sensitive laboratory species [24]. A persistent chironomid infestation had been observed intermittently in our facility since 2019, suggesting either continued internal proliferation or an unresolved route of introduction. This created a practical need for an objective, scalable, and time-efficient monitoring method. While convolutional neural networks (CNNs) have been used to identify adult Chironomidae [25, 26], recent advances in computer vision enable real-time quantification of larvae [27]. This study aimed to develop a simple AI-based desktop workflow to quantify infestation and evaluate two sanitation strategies in a *Xenopus laevis* aquatic system.

## Methods

### Animals and housing

The housing of *Xenopus laevis* frogs was in accordance with European (RL 2010/63EU) and German animal welfare legislation (5.1–231 5682/LMU/BMC/CAM) as previously described [28–30]. All frogs used in this study were sourced from the Core Facility Animal Models at the Biomedical Center of the LMU Munich. The animals had originally been obtained from Xenopus Express Inc. (Florida, USA) and had been housed at the facility for more than three months before the start of the study. Frogs were kept in groups of 10–15 animals per 87.3 L tank in a semi-closed system equipped with UV irradiation, water filtration, and automated water conditioning (Aqua Schwarz, Germany), with a total system volume of 1,250 L. Water was exchanged automatically twice daily, replacing approximately 130 L with fresh tap water. Tanks (99.5 × 58.5 × 25 cm) were enriched with dark shelters. Water temperature, pH, and conductivity were recorded daily. A 12:12 h light–dark cycle was applied. Health monitoring was conducted quarterly on 1–2 animals per system, and frogs were fed twice weekly (15 g fish feed per tank per meal; Teichsticks Premium, Interquell, Germany). Following completion of the study, none of the frogs were euthanized or released into the environment. All animals were returned to their original housing tanks and maintained under the same husbandry conditions as before the study. The frogs remained part of the facility’s research colony and were retained for future research use in accordance with institutional animal welfare regulations and approved protocols.

### AI-based detection and monitoring

To quantify adhesive-trapped Chironomids, we developed a custom Python-based desktop application (Chironomid Quantification Tool, CQT) integrating image preprocessing, tile-wise inference, result aggregation, and spreadsheet export into a standardized workflow. Adhesive strips were digitized at 1200 dpi and automatically partitioned into a non-overlapping 30 × 30 grid (900 tiles per scan). This tiling increased object scale and preserved local morphology for reliable detection. The detector was trained on a curated dataset of 100 annotated tiles (80 positive, 20 hard-negative). In total, 888 bounding boxes were annotated using a strict contour-based strategy, enclosing complete insect bodies only. Partial insects at tile boundaries were excluded from training to prevent learning truncated shapes. Object detection was performed with Ultralytics YOLO11n using a bounding-box-based approach (without segmentation). During inference, all tiles were processed independently, and only detections with a confidence score > 0.70 were retained to reduce false positives and duplicate counts. The model was able to separate closely adjacent or partially overlapping insects based on preserved body contours. The software was implemented in Python using Pillow (tiling), Ultralytics YOLO (inference), pandas (aggregation), Excel export, and PyQt5 (GUI). The application enabled loading scans, automated tiling, batch inference, and aggregation of detections into scan-level counts. Additionally, it generated annotated control images and exported tile-wise and total counts.

### Treatment and protocols

*Bacillus thuringiensis israelensis* (Bti; Culinex Tab plus, Culinex GmbH, Ludwigshafen, Germany) tablets containing 1000 international toxic units (ITU) per mg were applied according to the manufacturer’s recommendations. Two tablets per tank were pre-dissolved in 50 mL of water for 2 h before application. In total, 22 tablets (2 per tank; 0.55 g each) were applied to 1,250 L, resulting in a concentration of 9.68 × 10³ ITU L⁻¹. Treatment was performed weekly following prior system cleaning. Four sticky fly traps (Aeroxon Insect Control GmbH, Waiblingen, Germany) were attached to the dry surfaces of the filter towers and replaced weekly. As an alternative to Bti treatment, water parameters were modified to reduce larval development and to limit persistence of potential breeding sites. Filter towers and sludge filters were vacuumed twice weekly to remove sediment. In addition to automated twice-daily water exchange, an additional a manual water change was performed at noon from Monday to Friday. Both interventions were conducted over six consecutive weeks. Adult midge abundance was monitored using CQT-AI, while water parameters were recorded daily to ensure stable husbandry conditions.

## Results

**Fig. 1:**
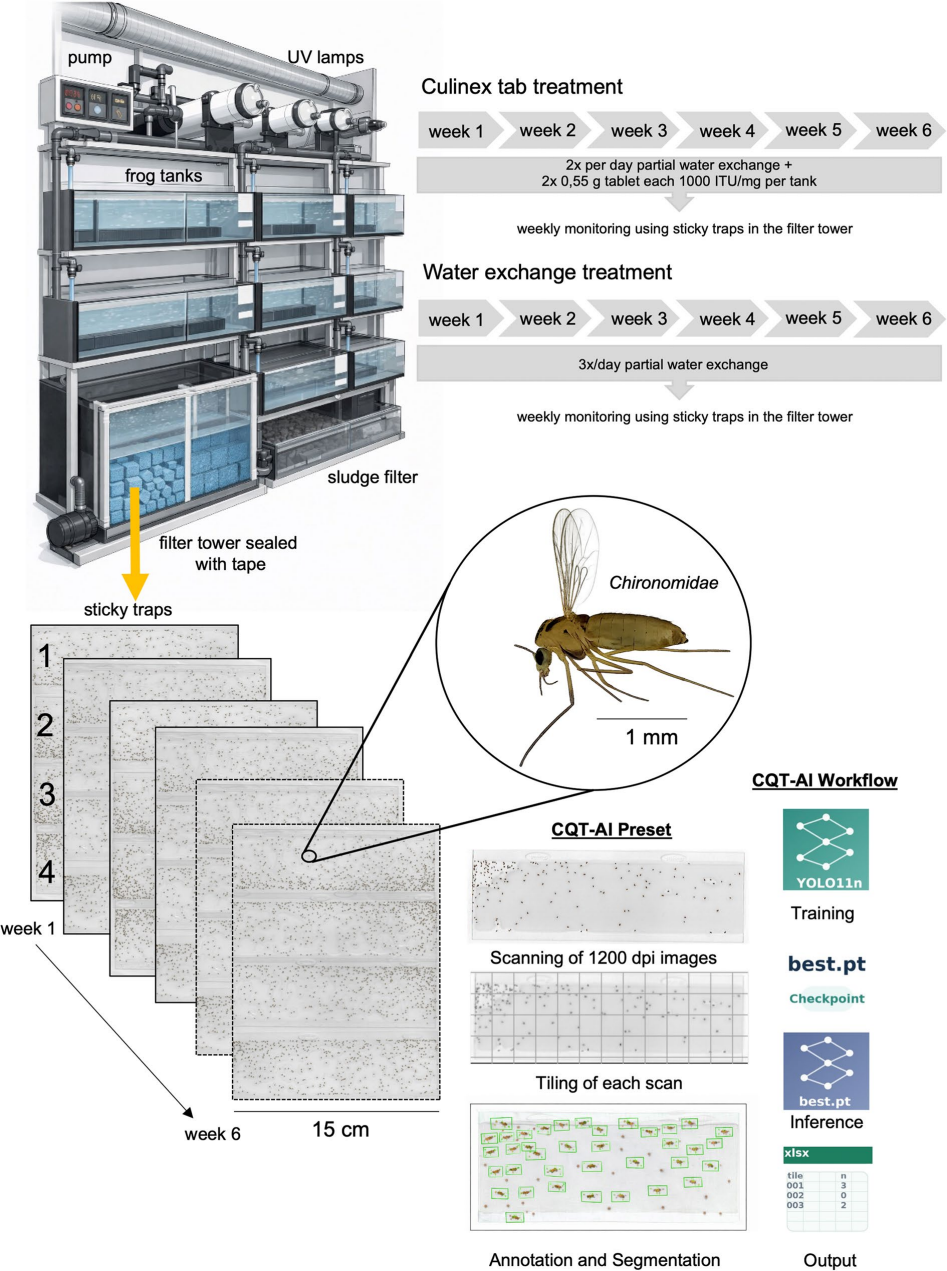
Experimental setup: The recirculating aquatic system consisted of frog tanks connected to pumps, UV lamps, and a sludge filter, with the filter tower sealed and equipped with sticky traps. Two intervention strategies were applied over six weeks: Bti treatment in combination with routine water exchange, and intensified water exchange alone. Sticky traps were collected weekly for chironomid monitoring. Representative trap images illustrate temporal changes in insect abundance. Samples were digitized, partitioned into image tiles, and analyzed using a YOLO11n-based object detection pipeline, including annotation, tile generation, automated detection, and count aggregation

**Fig. 2:**
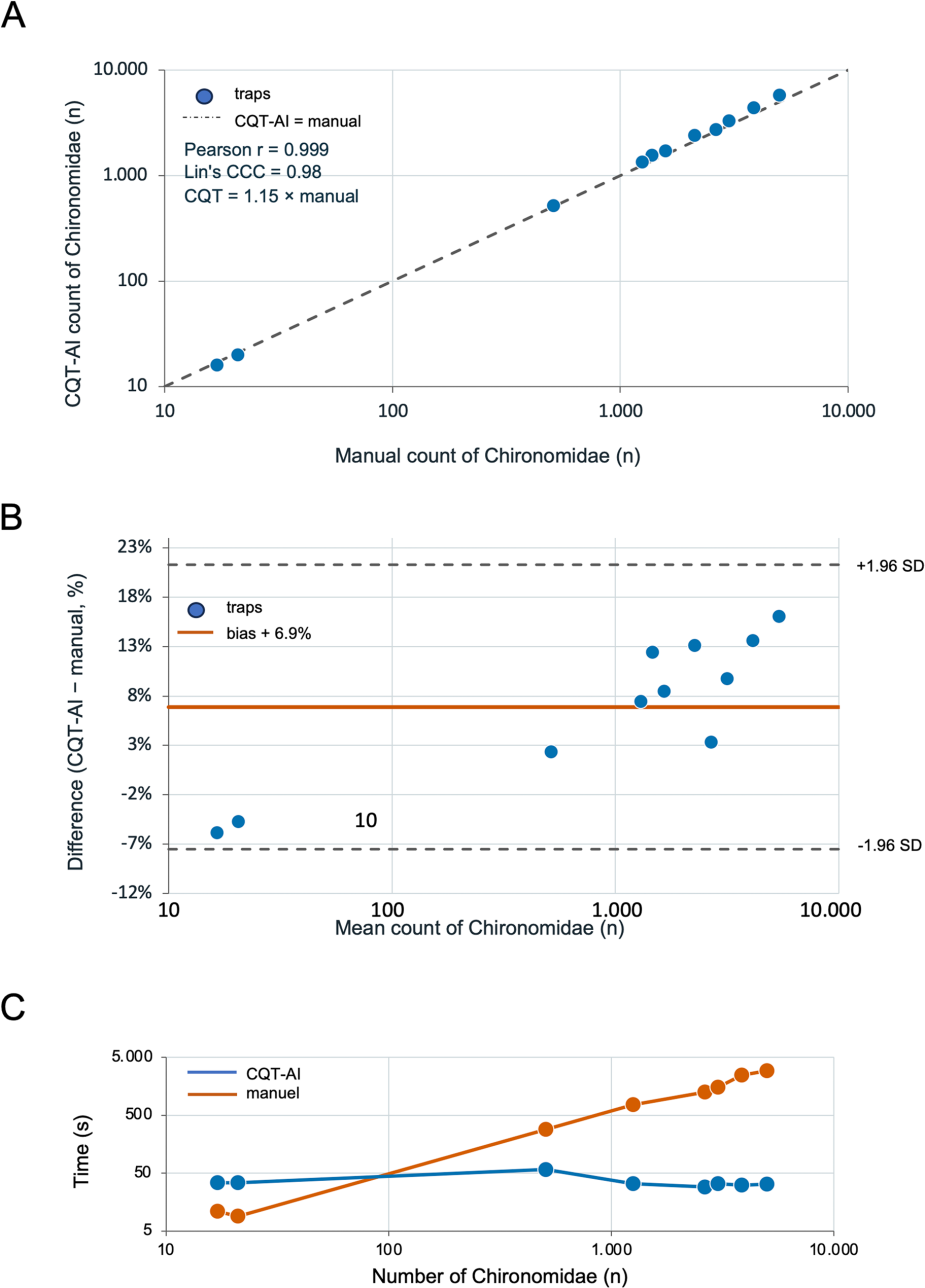
CQT-AI versus manual chironomid counts. **A** Count agreement across the full count range (Pearson *r* = 0.999; Lin’s CCC = 0.98; dashed line = identity). **B** Bland–Altman analysis of percentage differences (orange line = mean bias + 6.9%; dashed lines = 95% limits of agreement). **C** Counting time versus counted numbers of chironomids: CQT-AI runtime is essentially constant (33 s) and independent of the chironomid count, whereas manual counting scales at 56 s

To monitor infestation dynamics and evaluate treatment responses, the AI-based CQT-AI workflow was applied to weekly adhesive trap samples collected from the Xenopus laevis housing system (Fig. 1). High-resolution scans of the traps were analyzed using the tile-based object detection pipeline (Fig. 1). CQT-AI and manual counts were almost perfectly correlated (Pearson *r* = 0.999; Lin’s concordance correlation coefficient = 0.98) (Fig. 2A). The pipeline showed a small, systematic positive bias of + 6.9% on average that was consistent across the count range and negligible (± 1 *chironomid*) on sparsely populated traps (Fig. 2B). On traps of moderate *chironomid* density, automated counting was 23–91 times faster than manual counting; crucially, CQT-AI runtime was constant (33 s per scan) and independent of the number of flies, whereas manual counting scaled at around 0.56 s per chironomid (Fig. 2C).

**Fig. 3:**
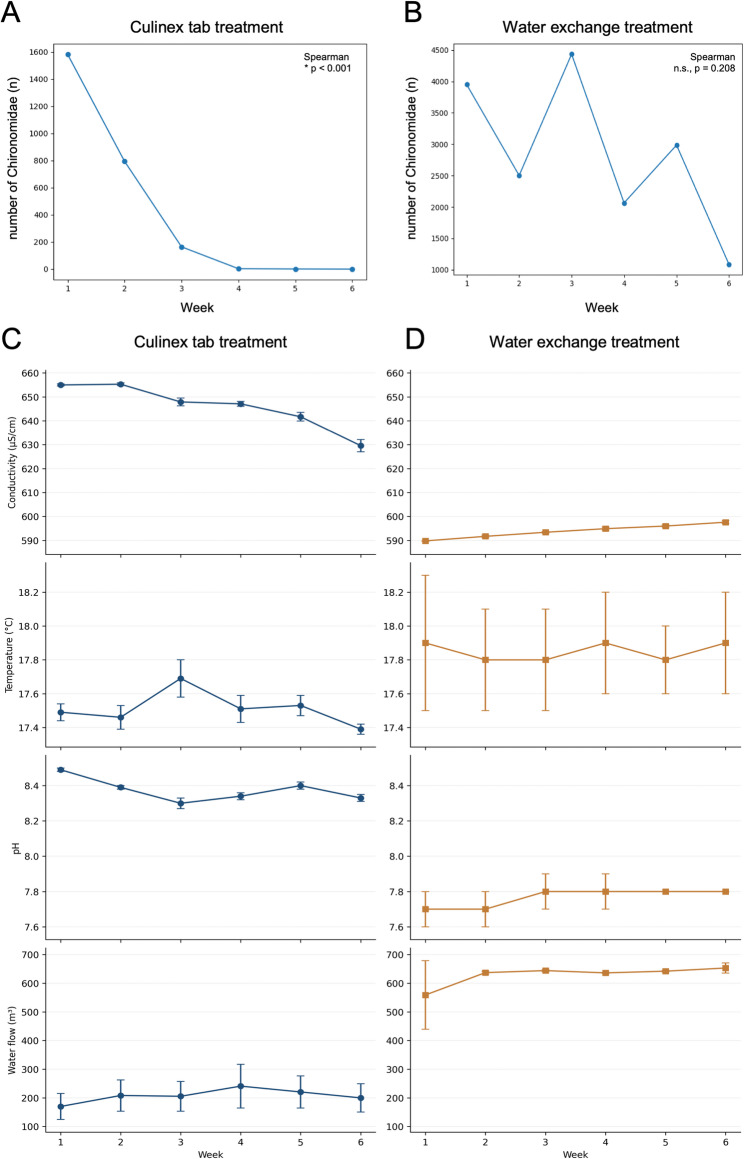
Comparison of treatment strategies. Effects of Bti treatment and intensified water exchange on adult *chironomid* abundance over six weeks. **A** *Chironomid* abundance under Bti treatment, showing a rapid and significant decline to near zero by week 4 (Spearman *p* < 0.001). **B** *Chironomid* abundance under intensified water exchange, showing temporal fluctuations throughout the experiment and non-significant decline over time (Spearman *p* < 0.208). **C–D** Environmental physicochemical parameters (conductivity, temperature, pH, and water flow; were measured weekly and compared using non-parametric test. All measured variables differed significantly between treatments (*p* < 0.01). Data are shown as mean ± SD; * *p* < 0.05, n.s. = not significant, Spearman-test (A/B) and U-Test C/D)

Using CQT-AI, baseline infestation levels were quantified for one week prior to application of *Bacillus thuringiensis israelensis* (Bti) tabs targeting larval development. The AI-assisted analysis detected a ~ 50% reduction in adult midge numbers after one week. A continued and significant decline was observed over weeks 2–3, and from weeks 4–6 no adult midges were detected on traps (Fig. 3A). Water parameters remained stable within standard husbandry ranges, and no adverse effects on animal health or experiments were observed (Fig. 3C). CQT-AI was further used to monitor infestation dynamics during intensified water exchange. The workflow detected an initial reduction in adult midge abundance after the first week (Fig. 3B), followed by a rebound in subsequent weeks, approaching baseline levels and showing a cyclical, statistically non-significant, decline–increase pattern. Although a reduction was again observed toward week 6, approximately 1,000 midges were still quantified by the AI-based analysis (Fig. 3B). All measured environmental parameters differed significantly between treatment groups (Mann-Whitney U test, *p* < 0.01). Conductivity and pH were significantly lower in water exchange treatment D, whereas temperature and water flow were significantly higher compared with Bti tab treatment (Fig. 3C and D). The strong reduction in conductivity and pH in treatment D is consistent with the additional water exchange performed during the experiment. Overall, the results demonstrate that CQT-AI enabled rapid, objective, and sensitive monitoring of infestation dynamics and treatment efficacy in the aquatic laboratory system.

## Discussion

Insect infestations in laboratory animal facilities represent a relevant hygienic concern because their effects on environmental quality, animal health, and experimental stability cannot be excluded. In the present case, the infestation occurred in an aquatic *Xenopus laevis* husbandry system and involved small flying insects morphologically consistent with non-biting midges *Chironomidae*, characterized by small body size, elongated legs, reduced or non-functional mouthparts, and narrow, non-scaled wings. These features are typical of non-biting midges and distinguish them from other dipteran families such as *Culicidae* [31]. Morphological traits suggest affiliation with the subfamily *Orthocladiinae*, which are typically small, inconspicuously colored midges lacking distinctive external markings. However, species-level identification was not confirmed by other methods. Literature on *Chironomidae* indicates that non-biting midges can proliferate in artificial aquatic systems, create nuisance problems, and act as reservoirs of aquatic bacteria [9, 10, 32]. Because *Chironomidae* were found almost exclusively in the filter and sludge towers of the recirculation system and were not observed in the housing tanks of the clawed frogs themselves, we assumed that the insects had no direct impact on the frogs’ wellbeing. However, it cannot be ruled out that insects may serve as reservoirs for pathogens such as *Aeromonas species (sp.)*. Such pathogens can pose a health risk to both frogs and humans [13, 33]. During sanitation measures, *Aeromonas* sp. were isolated from sludge tank water samples and from frog skin during routine health monitoring. It remains unclear whether this contamination was associated with the chironomid infestation or originated from the frogs themselves and was detected incidentally in asymptomatic animals. Nevertheless, the presence of adult midges caused considerable disruption to the facility and staff. To prevent spread to other barrier units, filter towers were sealed, and electric insect traps were used to control free-flying insects. Ultraviolet (UV) irradiation, commonly applied in aquatic systems, was employed for microbial control, particularly for inactivating bacteria, viruses, and protozoa [34]. However, its effectiveness is largely limited to microorganisms and is less reliable for larger aquatic organisms such as insect larvae. UV efficacy strongly depends on light penetration, which can be significantly reduced by turbidity, dissolved organic matter, and physical shielding [35]. Chironomid larvae typically inhabit sediments or construct protective tubes, thereby reducing direct UV exposure. In addition, some chironomid species exhibit remarkable tolerance to environmental stressors, including radiation, supported by efficient DNA repair mechanisms and enzyme activity [36]. Given the inadequate inactivation achieved by the technical measures installed in the facility, it was necessary to pursue therapeutic approaches to minimize the severity of the infestation, which was monitored by our self-designed CQT-AI. We tested the efficacy of a water quality stabilizing method alongside the widely used and commercially available biological larvicide containing *Bacillus thuringiensis israelensis* (Bti) [24]. A rapid and near-complete eradication of adult midges in the filter towers was achieved within six weeks of treatment. During the initial implementation in 2019, infestation levels were low at baseline, facilitating rapid control. Despite extensive root cause analyses, including microbiological feed assessments, no entry source could be identified. In contrast, infestation intensity during the second occurrence was more than fourfold higher, suggesting incomplete elimination of larvae within the system or re-infestation from an unidentified external source. As a second measure, we evaluated the effect of increased water exchange on temperature to reduce larval development rates and promote larval dispersal from the system. Additionally, sediment accumulation in sludge filters was minimized to limit potential breeding sites. Comparable sanitation approaches for *Chironomidae* in drinking water systems include reducing turbidity and temperature, as well as co-treatment with UV irradiation [37]. Increased daily water exchange rates led to decreased conductivity and pH, indicating dilution of dissolved ions and altered buffering capacity. These patterns are consistent with physicochemical dynamics in recirculating aquatic systems, where water renewal reduces ionic strength and affects alkalinity and pH stability. Although treatment-associated fluctuations are statistically significant, the observed differences in temperature and pH remained within biologically stable ranges and were therefore considered unlikely to substantially affect the frogs. Further, measured water parameters remained within recommended ranges for *Xenopus species* [38, 39]. Using the CQT-AI workflow, changes in adult *Chironomidae* abundance could be rapidly and objectively quantified over the six-week observation period, demonstrating the suitability of the system for monitoring infestation dynamics and treatment responses. Although increased water exchange resulted in a measurable reduction of adult individuals, targeted biocontrol using Bti-based formulations was identified by the AI-assisted analysis as significantly more effective than system-based intervention alone. Overall, the results of this pilot study demonstrate that CQT-AI is a fast and reliable tool for the detection and quantification of *Chironomidae*, enabling objective monitoring of control measures under laboratory conditions while generating visual documentation and spreadsheet-based outputs.

## Conclusion

CQT-AI enables accurate monitoring of infestation levels and treatment dynamics by reliably detecting adult *Chironomids*. Bti treatment led to a rapid and near-complete decline in midge abundance within three weeks, indicating high efficacy. In contrast, increased water exchange resulted only in moderate reductions with strong population fluctuations. Overall, the study highlights the potential of CQT-AI for monitoring *Chironomid* control in *Xenopus laevis* facilities.

### Strengths and limitations

This study demonstrates the suitability of the AI-driven CQT-AI workflow for unbiased, rapid, and accurate quantification of insect infestation, enabling objective monitoring and evaluation of control strategies in aquatic laboratory systems. To our knowledge, this is the first AI-assisted approach investigating Chironomidae monitoring and control in a *Xenopus laevis* laboratory facility. In this pilot study, two treatment strategies were applied primarily to evaluate the capability of CQT-AI to detect and quantify changes in infestation dynamics under different intervention conditions. However, direct comparison of treatment efficacy was limited by differing baseline infestation levels. Importantly, the AI-based analysis quantified only free-flying adult midges captured on sticky traps, while no data were collected on larval stages. It also remains unclear whether residual adult midges persisted after six weeks and contributed to re-infestation, and the original source of infestation could not be identified. A potential limitation of the present study is that adhesive traps were not only used for monitoring but may also have contributed to the removal of adult Chironomidae from the system. In a relatively small and enclosed facility, repeated trapping could theoretically influence population dynamics by reducing the number of reproducing adults. However, the traps represented a passive monitoring approach and targeted only a fraction of the free-flying adult population, while larval stages within the aquatic system remained unaffected. Therefore, trapping alone is unlikely to account for the pronounced decline observed following Bti treatment. Nevertheless, a potential contribution of continuous adult removal to population suppression cannot be excluded. Further studies are required to optimize Bti application protocols and combined intervention strategies, while also expanding and validating the AI-based workflow for robust long-term monitoring and eradication of Chironomidae in laboratory systems.

## Data Availability

The data that support the findings of this study are available from the corresponding author upon reasonable request. The code for the quantification AI is available via the following link: https://github.com/maxiimoa/CQT-AI.

## Abbreviations

AI: Artificial intelligence
Bti: Bacillus thuringiensis israelensis
cm: Centimeter
CQT: Chironomid Quantification Tool
FELASA: Federation of European Laboratory Animal Science Associations
g: Gram
GUI: Graphical user interface
h: Hour
ITU: International toxic units
L: Liter
mg: Milligram
min: Minutes
n: Number
p: Probability
pH: Potentia hydrogenii
s: Seconds
SD: Standard deviation
UV: Ultraviolet
µS: Micro-Siemens
YOLO: You only look once
